# Multimodal integration of serum DAPK1 and gastric myoelectrical activity reveals stage-specific correlates in Parkinson’s disease

**DOI:** 10.3389/fnagi.2026.1866257

**Published:** 2026-08-07

**Authors:** Zhining Li, Pei Lu, Can Li, Liangqun Rong, Zixuan Wu, Yumeng Liu, Yuyao Tian, Aiping Gong, Lishun Xiao, Chun-feng Liu

**Affiliations:** 1 Department of Neurology, The Second Affiliated Hospital of Xuzhou Medical University, Xuzhou, Jiangsu, China; 2 Department of Neurology and Clinical Research Center of Neurological Disease, The Second Affiliated Hospital of Soochow University, Suzhou, Jiangsu, China; 3 School of Public Health, Xuzhou Medical University, Xuzhou, Jiangsu, China; 4 Department of Biomedical Engineering, The Chinese University of Hong Kong, Shatin, Hong Kong SAR, China

**Keywords:** biomarker, DAPk1, electrogastrography, multimodal assessment, Parkinson’s disease, stage-specific correlates

## Abstract

**Objective:**

Integrated biomarkers for cross-sectional multidimensional assessment of Parkinson’s disease (PD) remain limited. This study examined associations between serum DAPK1, a systemic molecular marker, and peripheral gastric myoelectrical activity assessed by electrogastrography (EGG) in PD.

**Methods:**

In this cross-sectional study enrolling 203 participants, including 101 healthy controls (HC), 50 early-stage PD (EPD), and 52 mid-to-late-stage PD (LPD), we collected clinical data, serum DAPK1 levels, and EGG measurements. Stage-specific multivariable association frameworks incorporated age, gender, and BMI as key confounders. Statistical evaluation used likelihood ratio tests (LRT), Nagelkerke’s pseudo-R^2^, integrated discrimination improvement (IDI), and decision-curve analysis (DCA), with internal validation and optimism assessment using 1,000 refit bootstrap resamples.

**Results:**

For cross-sectional HC-versus-EPD assessment, multivariable analysis identified affective burden, higher DAPK1, and lower postprandial reaction area (PostRA) as independent correlates. The integrated E3 framework improved over the clinical-neuropsychological baseline (IDI = 0.072, *P* = 0.002), explaining 46.0% of variance (pseudo-R^2^ = 0.460) and achieving an apparent AUC of 0.851. For cross-sectional EPD-versus-LPD assessment, increased DAPK1, decreased preprandial mean amplitude (PreMA), and increased postprandial gastric rhythm (PostGR) were associated with more advanced disease status. The integrated S2 framework showed additional explanatory value (IDI = 0.219, *P* < 0.001), explaining 45.8% of variance (pseudo-R^2^ = 0.458) with an apparent AUC of 0.847. DCA suggested potential net benefit for the integrated frameworks within this cohort.

**Conclusion:**

Serum DAPK1 and peripheral EGG measures showed stage-specific cross-sectional associations in PD. Combining systemic DAPK1 and peripheral EGG data may support non-invasive multidimensional assessment of PD, but external validation and longitudinal studies are required before clinical implementation.

## 1 Introduction

Parkinson’s disease (PD) is an age-related neurodegenerative condition characterized by motor and non-motor manifestations (Hayes, 2019; Poewe et al., 2017; Tanner and Ostrem, 2024). Gastrointestinal (GI) dysfunction is common in PD and may occur early in the disease course (Fasano et al., 2015; Travagli et al., 2020; Anis et al., 2022; Konings et al., 2023; Park et al., 2025; Pasricha and Kulkarni, 2025; Yan et al., 2024); however, the presence of GI symptoms or gastric functional changes does not by itself establish a gastrointestinal origin of PD. Accurately characterizing GI dysregulation may help describe disease-related heterogeneity, but cross-sectional clinical studies should avoid causal or origin-of-disease inferences (Warnecke et al., 2022; Breen et al., 2019).

Electrogastrography (EGG) is a non-invasive technique used to record gastric myoelectrical activity (Wang et al., 2003; Sanders et al., 2024; Varghese et al., 2026a,b). Previous work has investigated EGG abnormalities in relation to PD-related gastric dysfunction and early-stage PD assessment (Anis et al., 2022; Oertel et al., 2024; Pasricha and Kulkarni, 2025; Simmonds et al., 2025); therefore, in the present study, EGG was treated as an established peripheral functional readout rather than as a novel disease-origin marker (Araki et al., 2021).

Death-associated protein kinase 1 (DAPK1) was evaluated from a different perspective. DAPK1 is a calcium/calmodulin-dependent serine/threonine kinase involved in apoptosis, autophagy, and neuroinflammatory signaling (Mansour et al., 2023; Soraci et al., 2024; Zhang et al., 2024). Experimental studies suggest that DAPK1 can phosphorylate alpha-synuclein at Ser129 and may contribute to alpha-synuclein aggregation under specific model conditions (Kim S. et al., 2019; Mifflin et al., 2020; Ding et al., 2025; Li et al., 2026); however, these experimental findings do not establish that circulating serum DAPK1 reflects gastrointestinal-origin pathology in human PD (Kelly et al., 2014; Kim N. et al., 2019; Shin and Chung, 2020).

Although structural and fluid biomarkers for PD, such as plasma neurofilament light chain and alpha-synuclein seeding assays, have been widely investigated (Halloway et al., 2022; Daponte et al., 2025; Tolosa et al., 2026), integrated approaches that evaluate systemic molecular changes together with peripheral gastric functional measures remain limited. The rationale for integrating serum DAPK1 and EGG in this study was not to infer a gastrointestinal origin or a causal gut-brain-axis mechanism, but to test whether an exploratory circulating molecular correlate and an established gastric functional readout provide complementary cross-sectional information across PD stage groups. Using sequential multivariable modeling, we evaluated stage-specific associations while explicitly avoiding causal, longitudinal staging, or disease-origin claims.

## 2 Materials and methods

### 2.1 Participants

A cross-sectional study was conducted involving participants recruited from the Department of Neurology at the Second Affiliated Hospital of Xuzhou Medical University between March 2021 and June 2025. The PD cohort included individuals diagnosed according to the 2015 MDS clinical diagnostic criteria who were on a stable regimen of dopaminergic therapy (Postuma et al., 2015). To facilitate severity stratification modeling, the PD cohort was prospectively dichotomized into early-stage PD (EPD) and mid-to-late-stage PD (LPD) based on the Hoehn and Yahr (H&Y) staging scale (H&Y ≤ 2.5 for EPD, and H&Y ≥ 3 for LPD).

Exclusion criteria for PD patients were established to minimize confounding factors: (i) presence of severe systemic disorders, oncological diseases, acute inflammatory conditions, or inability to cooperate; (ii) comorbid conditions altering gastric myoelectrical activity (e.g., diabetes mellitus, functional dyspepsia, *H. pylori* infection, prior abdominal surgery, or thyroid dysfunction); and (iii) use of medications altering GI motility (e.g., prokinetics, antispasmodics), antibiotics, or probiotics within 1 week prior to examination.

Healthy controls (HC) consisted of age-matched individuals between 45 and 80 years old free from acute/chronic medical conditions, abdominal surgery, and GI-modulating medications. The final analysis included data from 102 PD patients and 101 HC. The study was approved by the ethics committee of the Second Affiliated Hospital of Xuzhou Medical University (No. 2021-021701).

### 2.2 Clinical and neuropsychological assessments

Prior to the acquisition of electrophysiological data, all patients with PD underwent systematic clinical evaluations.

Participants were assessed while in their dopaminergic “ON” state. To guarantee cultural appropriateness, linguistic accuracy, and data integrity, we employed officially translated and psychometrically validated Chinese versions of all assessment instruments. Motor symptom severity was quantified using the Movement Disorder Society Unified Parkinson’s Disease Rating Scale Part III (MDS-UPDRS-III) (Goetz et al., 2008; Guo et al., 2023). Non-motor manifestations, particularly gastrointestinal autonomic dysfunction, were comprehensively evaluated via the Non-Motor Symptoms Scale (NMSS) and its specific gastrointestinal subscale (NMSS-GI) (Wang et al., 2009). Global cognitive performance was determined by retrospectively extracting scores from routine clinical evaluations archived in the hospital’s electronic medical record (EMR) system; these records included Montreal Cognitive Assessment (MoCA) scores. Furthermore, neuropsychiatric status was assessed using the Hamilton Anxiety Rating Scale (HAMA) and the Hamilton Depression Rating Scale (HAMD). Levodopa Equivalent Daily Dose (LEDD) values were computed following established conversion guidelines.

### 2.3 Serum DAPK1 assay

Fasting venous blood (5 mL) was collected in coagulation tubes. After clotting for 30 min at room temperature, samples were centrifuged at 1000 × *g* for 20 min at 4 °C. Serum aliquots were stored at −80 °C without undergoing freeze-thaw cycles. Due to the limited volume of clinical serum available for this assay, serum DAPK1 concentrations were quantified in a single well per specimen using a Human DAPK1 ELISA Kit (Jianglai Biotechnology, Shanghai, China) following the manufacturer’s protocol. To prevent assessment bias, laboratory technicians performing the assays were blinded to the clinical diagnosis and grouping of the samples. According to the kit datasheet, the manufacturer-reported intra-assay and inter-assay coefficients of variation were less than 10.0%; these values describe kit-level performance and should not be interpreted as sample-level repeatability in this cohort.

### 2.4 Electrogastrography (EGG) recording and signal processing

Gastrointestinal myoelectrical activity was recorded using an electrogastrogram (EGG) device (XDJ-S8, Hefei Kaili Co., Hefei, China). All participants were instructed to abstain from alcohol, as well as spicy, greasy, or irritating foods, for 72 h preceding the recording. Before the morning assessments, participants maintained a clear liquid diet and fasted for over 8 h. To minimize medication-induced motility artifacts, PD patients were evaluated in a practically defined OFF state, withholding all antiparkinsonian medications for at least 12 h prior to the procedure. Furthermore, vigorous physical exertion, smoking, and mentally stressful activities were prohibited. All examinations were conducted in a dedicated, quiet room. Participants were instructed to rest comfortably in a supine position, maintain a stable emotional state, and avoid any movement, talking, or sleeping during the entire recording period. Four gastric electrodes (Hanjie Co., Ltd., Shanghai, China) were placed on the abdominal skin as follows: (i) electrode one, 1 cm above the midpoint between umbilicus and xiphoid and 3–5 cm to the left of the midline; (ii) electrode two, the upper third quarter point from umbilicus to xiphoid; (iii) electrode three, the first quarter point from umbilicus to xiphoid; (iv) electrode four, 2–4 cm to the right of the midpoint between umbilicus and xiphoid. This spatial configuration is in accordance with standard published electrogastrography placement protocols (Wang et al., 2003; Peng et al., 2021). Although the XDJ-S8 is an 8-channel electrogastrography system capable of recording broader gastrointestinal activity, the primary focus of this study was specifically on gastric myoelectrical signals. Consequently, only data from channels 1–4 (covering the stomach) were extracted and analyzed, while recordings from the lower gastrointestinal tract (channels 5–8) were excluded from the current analysis. The reference electrode was placed on the medial side of the wrist, and the grounding electrode was placed on the ipsilateral ankle. The skin at the electrode placement sites was carefully wiped with anhydrous (or 95%) alcohol-cotton balls for degreasing. After a 20-min preprandial EGG recording, participants were instructed to take standard food containing 50 g of bread and 400 mL of water in 10 min. This standardized low-calorie carbohydrate load was specifically selected to ensure high tolerability, prevent aspiration, and accommodate the varying degrees of dysphagia and delayed gastric emptying commonly observed in advanced Parkinson’s disease patients (Bahat et al., 2020). To control for the confounding effects of food intake on postprandial electrophysiological measures, a 10-min standardized meal protocol was strictly implemented. The final sample included 101 healthy controls, 50 early-stage PD and 52 mid-to-late-stage PD participants, all fully compliant with the unified feeding procedure to ensure consistent postprandial physiological stimulation. Subsequently, an additional 20-min postprandial recording was acquired.

Electrogastrography recordings were performed at a sampling rate of 1 Hz. A bandpass filter was applied, with low and high cutoff frequencies of 0.008 Hz and 0.15 Hz, to eliminate background noise such as heartbeat-related interference. To ensure signal reliability, raw EGG data were visually inspected by two independent researchers blinded to clinical status. Discrepancies in artifact identification were resolved through discussion. Segments containing high-frequency noise from body movement, respiratory interference, or baseline drift outside physiological ranges were manually excluded. Following visual inspection and manual artifact rejection, an average of approximately 8.5% of the raw recording duration was excluded per participant, and no included participant exceeded a 10% exclusion threshold. Spectral analysis was performed on the remaining artifact-free segments individually, and the resultant power spectra were averaged per participant to avoid boundary artifacts. The following parameters were computed for both the 20-min preprandial and 20-min postprandial recordings: (i) mean amplitude (MA, e.g., PreMA and PostMA), calculated as the average peak-to-trough voltage (μV) of time-domain waveforms; (ii) mean frequency (cpm), defined as the power-weighted average frequency within the physiological band (0.5–9.0 cpm), calculated using the following formula:


$$\mathrm{Mean}\,\mathrm{Frequency}=\frac{\Sigma \,{\text{P}}_{\text{i}}\times {\text{f}}_{\text{i}}}{\Sigma \,{\text{P}}_{\text{i}}}$$

where Pi was the power density at frequency bin fi. This parameter reflected the center of gravity of the power spectrum and provided a robust estimation of the average pacemaker rate, minimizing the impact of transient unstable peaks often observed in gastroparesis. (iii) Gastric Rhythm (GR, e.g., PreGR and PostGR): representing the percentage of dysrhythmia (%), which is the proportion of recording time with irregular waveforms (tachygastria and bradygastria). (iv) Reaction area (RA, e.g., PreRA and PostRA): representing the power response area (μV⋅s), calculated as the integral of amplitude over time, quantifying overall electrical activity.

### 2.5 Statistical analysis

Statistical analyses were performed using R software (version 4.5.1). Continuous variables are presented as mean ± standard deviation, and categorical variables as frequency (percentage). Group comparisons were conducted using Student’s *t*-test, Welch’s *t*-test, analysis of variance (ANOVA), or the χ^2^ test, as appropriate. For multi-group comparisons (HC vs. EPD vs. LPD), significant main effects in ANOVA were followed by *post hoc* pairwise comparisons using Tukey’s Honestly Significant Difference (HSD) test. For categorical variables, multiple testing was adjusted using the Bonferroni correction. Partial Spearman’s rank correlation adjusted for potential confounders was used to evaluate clinical associations, and *P*-values were adjusted using the Benjamini-Hochberg false discovery rate (FDR) procedure. To represent overall neuropsychiatric affective burden, Mood Score was calculated as the sum of the raw HAMA and HAMD scores. Global cognition (MoCA) was evaluated as an independent continuous variable in subsequent analyses.

To identify stage-specific cross-sectional correlates, variables significant in univariate screening (*P* < 0.05) were entered into backward stepwise multivariable binary logistic regression with prespecified criteria (entry threshold *P* < 0.05; removal threshold *P* > 0.10). Gender was treated as a binary dummy variable and recoded from the original data as Female = 0 and Male = 1, with Female serving as the reference group. Age, gender, and BMI were entered as forced covariates to control prespecified demographic confounding. This approach controls basic confounding but does not by itself eliminate overfitting; therefore, candidate predictors were restricted (Vittinghoff and McCulloch, 2007), multicollinearity was screened using a conventional VIF > 5 exclusion threshold, and internal validation was performed. The models’ overall fit and explained variance were assessed using the likelihood ratio test (LRT) and Nagelkerke’s pseudo-R^2^, respectively. Discriminative explanatory performance was quantified by the area under the receiver operating characteristic curve (AUC). Internal validation and optimism assessment were performed using 1,000 refit bootstrap resamples to estimate bootstrap mean AUCs, 95% confidence intervals, and optimism-corrected AUCs. Integrated discrimination improvement (IDI) was calculated to assess incremental explanatory capacity between sequential frameworks. Decision-curve analysis (DCA) was used to evaluate potential net benefit across threshold probabilities within this cohort. All AUC and DCA findings were interpreted as internally evaluated cross-sectional model performance rather than evidence of external validation, longitudinal prediction, or ready clinical implementation. All statistical tests were two-sided, with significance set at α = 0.05.

## 3 Results

### 3.1 Baseline characteristics and univariate feature selection

This study included 203 participants (101 HC, 50 EPD, and 52 LPD). Overall comparisons showed no significant between-group differences in sex distribution, age, BMI, or educational duration (all *P* > 0.05, Table 1). To ensure a highly rigorous control of potential baseline demographic confounding, age, gender, and BMI were explicitly designated as prespecified forced covariates in all downstream multivariable association frameworks (Table 1).

**TABLE 1:** Baseline characteristics, serum DAPK1, and electrogastrography parameters of the study cohort.

Variable	HC (*n* = 101)	EPD (*n* = 50)	LPD (*n* = 52)	Overall *P*	*P*1	*P*2	*P*3
Gender: Female	50 (49.50)	24 (48.00)	24 (46.15)	0.925	0.999	0.824	1.000
Male	51 (50.50)	26 (52.00)	28 (53.85)	–	–	–	–
Age (years)	67.29 ± 8.89	67.90 ± 7.82	70.69 ± 8.56	0.064	–	–	–
BMI (kg/m^2^)	22.68 ± 1.73	23.15 ± 1.47	22.73 ± 1.84	0.261	0.084	0.857	0.210
Educational duration (years)	6.86 ± 3.62	6.96 ± 3.12	6.56 ± 2.40	0.798	0.863	0.536	0.469
Disease duration (years)	–	3.68 ± 2.57	5.40 ± 2.89	–	–	–	0.002
LEDD (mg/day)	–	409.46 ± 178.27	477.15 ± 210.52	–	–	–	0.082
Serum DAPK1 (ng/mL)	1.46 ± 1.42	2.37 ± 1.94	3.48 ± 2.62	<0.001	0.004	<0.001	0.017
HAMA score	6.17 ± 1.78	9.22 ± 4.94	9.31 ± 3.18	<0.001	<0.001	<0.001	0.916
HAMD score	3.72 ± 1.83	7.36 ± 5.01	7.10 ± 3.92	<0.001	<0.001	<0.001	0.768
Global-Cog (MoCA)	23.11 ± 3.01	19.94 ± 4.65	19.23 ± 3.13	<0.001	<0.001	<0.001	0.371
MDS-UPDRS total score	–	40.60 ± 19.15	75.58 ± 22.23	–	–	–	<0.001
MDS-UPDRS III score	–	22.08 ± 11.77	51.33 ± 15.80	–	–	–	<0.001
NMSS total score	–	39.18 ± 22.22	53.56 ± 25.95	–	–	–	0.003
NMSS GI domain score	–	4.14 ± 2.35	7.65 ± 5.63	–	–	–	<0.001
PreMA (μV)	218.51 ± 89.93	215.99 ± 89.33	157.66 ± 64.14	<0.001	0.871	<0.001	<0.001
PostMA (μV)	293.97 ± 118.23	216.65 ± 92.46	191.22 ± 86.08	<0.001	<0.001	<0.001	0.154
PreMF (cpm)	3.62 ± 0.33	3.54 ± 0.35	3.56 ± 0.34	0.335	0.194	0.292	0.805
PostMF (cpm)	3.53 ± 0.27	3.58 ± 0.26	3.55 ± 0.32	0.510	0.230	0.588	0.633
PreGR (%)	22.34 ± 3.10	21.85 ± 3.86	23.11 ± 3.50	0.167	0.441	0.180	0.087
PostGR (%)	21.38 ± 3.41	21.59 ± 3.99	23.24 ± 3.22	0.007	0.749	0.001	0.024
PreRA (μV⋅s)	80.22 ± 32.36	77.52 ± 33.04	58.56 ± 23.77	<0.001	0.635	<0.001	0.001
PostRA (μV⋅s)	105.77 ± 42.40	78.10 ± 32.96	69.90 ± 31.68	<0.001	<0.001	<0.001	0.204

Continuous variables are presented as mean ± SD, and categorical variables as *n* (%). Overall *P*-values were calculated using one-way ANOVA for continuous variables and chi-square tests for categorical variables. Pairwise comparisons were performed using Welch *t*-tests for continuous variables and chi-square tests for categorical variables. P1 compares HC with EPP; P2 compares HC with LPD; P3 compares EPP with LPD. Variables not applicable to HC are shown as en dash (–). HC, healthy controls; PD, Parkinson’s disease; EPP, early-stage Parkinson’s disease; LPD, mid-to-late-stage Parkinson’s disease; BMI, body mass index; LEPP, Levodopa Equivalent Daily Dose; DAPK1, death-associated protein kinase 1; MA, mean amplitude; MF, mean frequency; GR., gastric rhythm; RA, reaction area.

In the HC-versus-EPD assessment, univariate analysis revealed significant between-group differences in cognition (MoCA), affective scores (HAMA and HAMD), serum DAPK1, and postprandial gastric parameters (PostMA and PostRA). For EPD-versus-LPD assessment, significant differences were observed in disease duration, serum DAPK1, and selected pre- and postprandial EGG markers (PreRA, PreMA, and PostGR) (all *P* < 0.05). Cognitive-affective scores and LEDD did not differ significantly between PD stages.

Serum DAPK1 concentrations differed significantly across groups and increased from HC to EPD and LPD, with significant pairwise differences among all three groups (overall *P* < 0.001; HC vs. EPD, *P* = 0.004; HC vs. LPD, *P* < 0.001; EPD vs. LPD, *P* = 0.017; Table 1). Electrophysiologically, postprandial mean amplitude was significantly lower in both EPD and LPD than in HC (both *P* < 0.001). Preprandial mean amplitude and preprandial reaction area were significantly reduced mainly in LPD compared with both HC and EPD, while PostGR was higher in LPD than in HC and EPD (Table 1).

### 3.2 Correlations between biomarkers and clinical scales

After adjustment for age, gender, BMI, education, LEDD, and disease duration, serum DAPK1 levels showed positive partial correlations with NMSS-Total (*r* = 0.36, *P*FDR = 0.009) and NMSS-GI scores (*r* = 0.36, *P*FDR = 0.009), while remaining independent of motor and cognitive scales. Electrophysiologically, PreMA showed a negative partial correlation with MDS-UPDRS-III motor scores (*r* = −0.31, *P*FDR = 0.041), suggesting that basal gastric myoelectrical tone was modestly associated with motor severity (Table 2). Although statistically significant, these correlations showed modest effect sizes (| r| < 0.4), indicating partial concordance rather than parallel disease progression.

**TABLE 2:** Partial Spearman correlations between serum DAPK1/EGG parameters and clinical measures in patients with Parkinson’s disease.

Parameter	Clinical measure	*N*	r	*P*-value	FDR-adjusted *P*
Serum DAPK1 (ng/mL)	MDS-UPDRS total score	102	0.20	0.052	0.253
Serum DAPK1 (ng/mL)	MDS-UPDRS III score	102	0.20	0.057	0.256
Serum DAPK1 (ng/mL)	NMSS total score	102	**0.36**	**<0.001**	**0.009**
Serum DAPK1 (ng/mL)	NMSS GI domain score	102	**0.36**	**<0.001**	**0.009**
Serum DAPK1 (ng/mL)	Global-Cog (MoCA)	102	0.06	0.547	0.766
Serum DAPK1 (ng/mL)	HAMA score	102	−0.02	0.870	0.929
Serum DAPK1 (ng/mL)	HAMD score	102	−0.07	0.500	0.716
PreMA (μV)	MDS-UPDRS total score	102	−0.24	0.018	0.194
PreMA (μV)	MDS-UPDRS III score	102	−**0.31**	**0.002**	**0.041**
PreMA (μV)	NMSS total score	102	−0.22	0.035	0.249
PreMA (μV)	NMSS GI domain score	102	−0.26	0.009	0.121
PreMA (μV)	Global-Cog (MoCA)	102	0.09	0.405	0.683
PreMA (μV)	HAMA score	102	0.09	0.408	0.683
PreMA (μV)	HAMD score	102	0.05	0.616	0.808
PostMA (μV)	MDS-UPDRS total score	102	−0.14	0.181	0.497
PostMA (μV)	MDS-UPDRS III score	102	−0.19	0.071	0.282
PostMA (μV)	NMSS total score	102	−0.12	0.240	0.631
PostMA (μV)	NMSS GI domain score	102	−0.20	0.048	0.253
PostMA (μV)	Global-Cog (MoCA)	102	0.15	0.137	0.430
PostMA (μV)	HAMA score	102	0.09	0.373	0.683
PostMA (μV)	HAMD score	102	0.02	0.851	0.925
PreMF (cpm)	MDS-UPDRS total score	102	−0.08	0.446	0.687
PreMF (cpm)	MDS-UPDRS III score	102	−0.01	0.918	0.933
PreMF (cpm)	NMSS total score	102	−0.09	0.375	0.683
PreMF (cpm)	NMSS GI domain score	102	−0.05	0.629	0.808
PreMF (cpm)	Global-Cog (MoCA)	102	0.10	0.342	0.683
PreMF (cpm)	HAMA score	102	−0.05	0.662	0.809
PreMF (cpm)	HAMD score	102	−0.08	0.447	0.687
PostMF (cpm)	MDS-UPDRS total score	102	−0.07	0.473	0.693
PostMF (cpm)	MDS-UPDRS III score	102	−0.08	0.423	0.683
PostMF (cpm)	NMSS total score	102	−0.01	0.898	0.929
PostMF (cpm)	NMSS GI domain score	102	−0.03	0.752	0.894
PostMF (cpm)	Global-Cog (MoCA)	102	0.23	0.026	0.238
PostMF (cpm)	HAMA score	102	−0.05	0.624	0.808
PostMF (cpm)	HAMD score	102	0.03	0.773	0.895
PreGR (%)	MDS-UPDRS total score	102	0.01	0.899	0.929
PreGR (%)	MDS-UPDRS III score	102	0.02	0.812	0.897
PreGR (%)	NMSS total score	102	0.12	0.259	0.653
PreGR (%)	NMSS GI domain score	102	0.09	0.359	0.683
PreGR (%)	Global-Cog (MoCA)	102	0.11	0.294	0.675
PreGR (%)	HAMA score	102	0.02	0.810	0.897
PreGR (%)	HAMD score	102	<0.01	0.973	0.973
PostGR (%)	MDS-UPDRS total score	102	0.18	0.077	0.287
PostGR (%)	MDS-UPDRS III score	102	0.20	0.052	0.253
PostGR (%)	NMSS total score	102	−0.08	0.466	0.693
PostGR (%)	NMSS GI domain score	102	−0.03	0.781	0.895
PostGR (%)	Global-Cog (MoCA)	102	−0.06	0.565	0.773
PostGR (%)	HAMA score	102	0.08	0.416	0.683
PostGR (%)	HAMD score	102	0.05	0.642	0.809
PreRA (μVs)	MDS-UPDRS total score	102	−0.21	0.040	0.249
Pre RA (μVs)	MDS-UPDRS III score	102	−0.26	0.010	0.121
PreRA (μVs)	NMSS total score	102	−0.17	0.103	0.341
PreRA (μVs)	NMSS GI domain score	102	−0.21	0.037	0.249
PreRA (μVs)	Global-Cog (MoCA)	102	0.08	0.411	0.683
PreRA (μVs)	HAMA score	102	0.11	0.300	0.675
PreRA (mVs)	HAMD score	102	0.10	0.318	0.683
PostRA (μVs)	MDS-UPDRS total score	102	−0.14	0.167	0.478
Post RA (μVs)	MDS-UPDRS III score	102	−0.18	0.083	0.290
PostRA (μVs)	NMSS total score	102	−0.11	0.273	0.662
PostRA (μVs)	NMSS GI domain score	102	−0.18	0.072	0.282
PostRA (μVs)	Global-Cog (MoCA)	102	0.15	0.147	0.442
PostRA (μVs)	HAMA score	102	0.09	0.361	0.683
PostRA (μVs)	HAMD score	102	0.04	0.668	0.809

Partial Spearman correlation coefficients were adjusted for age, gender, BMI, educational duration, disease duration, and LEDD. FDR-adjusted *P*-values were calculated using the Benjamini-Hochberg method. Bold values indicate FDR-adjusted *P* < 0.05. EGG, electrogastrography; DAPK1, death-associated protein kinase 1; MDS-UPDRS, Movement Disorder Society Unified Parkinson’s Disease Rating Scale; NMSS, Non-Motor Symptoms Scale; MoCA, Montreal Cognitive Assessment; HAMA, Hamilton Anxiety Rating Scale; HAMP, Hamilton Depression Rating Scale.

### 3.3 Multivariable association framework for cross-sectional HC-versus-EPD assessment

To minimize multicollinearity, PostRA was prioritized for final inclusion after univariate screening and collinearity assessment (Supplementary Table 1). The multivariable association framework was developed incrementally: from demographic covariates (E1), to clinical-neuropsychological factors (E2), and then to the integrated E3 framework incorporating serum DAPK1 and PostRA (Supplementary Table 2).

**FIGURE 1:**
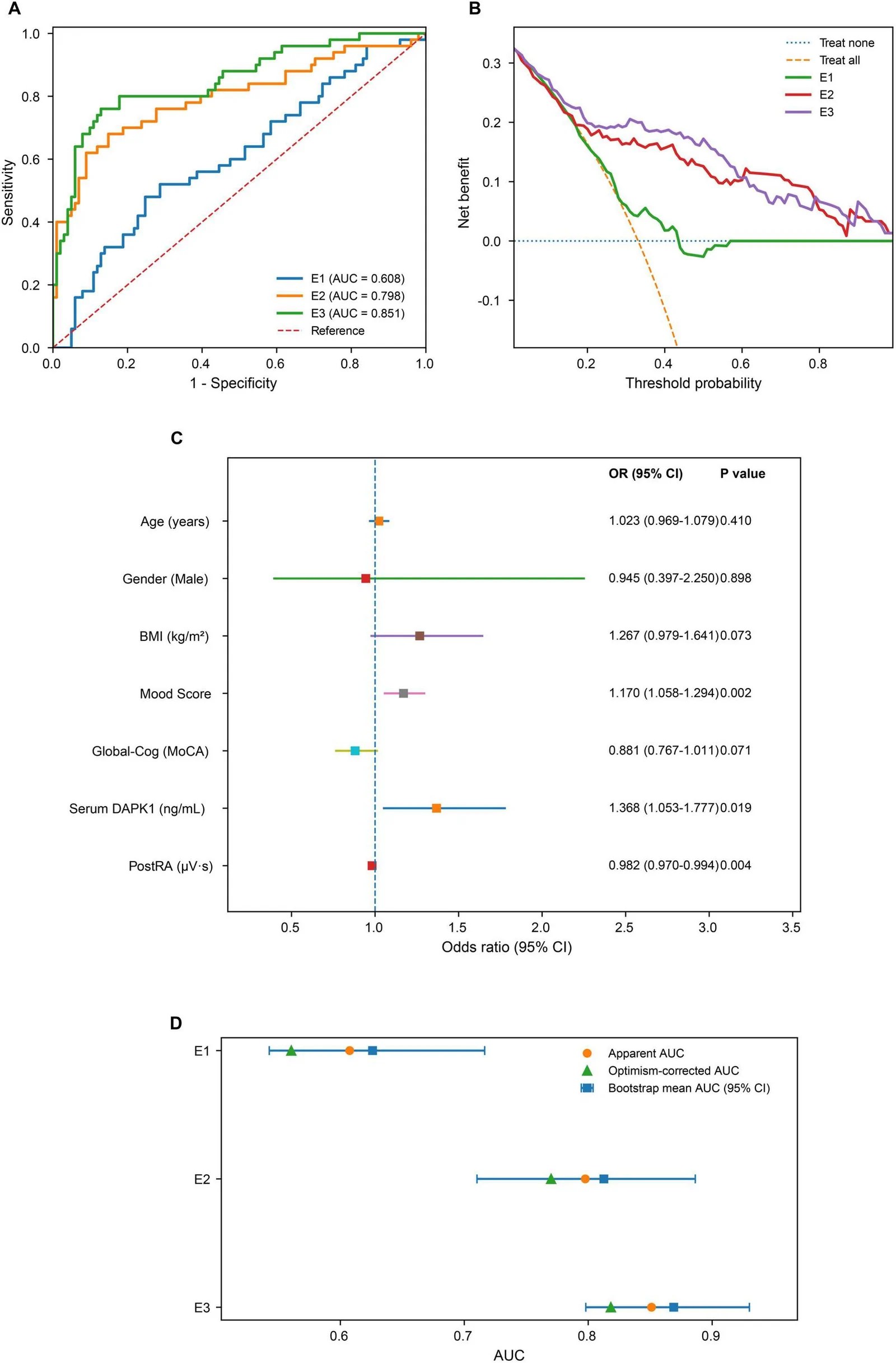
Performance and internal validation of the stepwise association frameworks for cross-sectional HC-versus-EPD assessment. **(A)** Receiver operating characteristic (ROC) curves comparing the clinical baseline framework (E1), the clinical-neuropsychological framework (E2), and the integrated multimodal framework (E3) for cross-sectional differentiation between healthy controls (HC) and early-stage Parkinson’s disease (EPD). E1 included age, gender, and body mass index (BMI); E2 additionally included Mood Score and Global-Cog (MoCA); and E3 further incorporated serum DAPK1 and postprandial reaction area (PostRA). The apparent AUCs were 0.608, 0.798, and 0.851 for E1, E2, and E3, respectively. **(B)** Decision-curve analysis (DCA) of E1, E2, and E3 across threshold probabilities. The E3 framework suggested a higher potential net benefit than the comparison frameworks across selected threshold ranges. **(C)** Forest plot of the final integrated E3 framework, showing odds ratios (ORs), 95% confidence intervals (CIs), and *P*-values for the retained covariates. Mood Score was calculated as the sum of HAMA and HAMD scores. **(D)** Internal validation and optimism assessment displaying the apparent AUC, optimism-corrected AUC, and bootstrap mean AUC with 95% confidence intervals for E1, E2, and E3 frameworks. HC, healthy controls; EPD, early-stage Parkinson’s disease; BMI, body mass index; MoCA, Montreal Cognitive Assessment; DAPK1, death-associated protein kinase 1; PostRA, postprandial reaction area; HAMA, Hamilton Anxiety Rating Scale; HAMD, Hamilton Depression Rating Scale; ROC, receiver operating characteristic; DCA, decision-curve analysis; AUC, area under the curve; OR, odds ratio; CI, confidence interval.

The integrated E3 framework showed improved explanatory performance compared with the preceding frameworks, with a Nagelkerke pseudo-R^2^ of 0.460 and an apparent AUC of 0.851 (Figure 1A). DCA suggested potential net benefit for the E3 framework across selected threshold ranges within this cohort (Figure 1B). Within this multivariable context, elevated DAPK1, reduced PostRA, and higher Mood Score were retained as independent correlates of EPD status (all *P* < 0.05, Figure 1C and Table 3). LRT and IDI analyses indicated that adding serum DAPK1 and PostRA provided incremental explanatory value over the clinical-neuropsychological baseline (LRT *P* < 0.001; IDI = 0.072, *P* = 0.002; Supplementary Table 2). Internal validation using 1,000 refit bootstrap resamples yielded a bootstrap mean AUC of 0.863 (95% CI: 0.791–0.925) and an optimism-corrected AUC of 0.823 (Figure 1D, Supplementary Figure 1A and Supplementary Table 2).

**TABLE 3:** Final integrated multivariable association model for cross-sectional HC-versus-EPD assessment.

Predictor	OR (95% CI)	*P*-value
Age (years)	1.023 (0.969–1.079)	0.410
Gender (Male)	0.945 (0.397–2.250)	0.898
BMI (kg/m^2^)	1.267 (0.979–1.641)	0.073
Mood Score	1.170 (1.058–1.294)	0.002
Global-Cog (MoCA)	0.881 (0.767–1.011)	0.071
Serum DAPK1 (ng/mL)	1.368 (1.053–1.777)	0.019
PostRA (μV⋅s)	0.982 (0.970–0.994)	0.004

Values are odds ratios (ORs), 95% confidence intervals (CIs), and *P*-values from the final integrated logistic regression model (E3). Gender was recoded before modeling as Female = 0 and Male = 1; Female served as the reference group. Mood Score was calculated as the sum of raw HAMA and HAMP scores. ORs for continuous variables represent the change in odds per one-unit increase. HC, healthy controls; EPP, early-stage Parkinson’s disease; BMI, body mass index; DAPK1, death-associated protein kinase 1; PostRA, postprandial reaction area.

### 3.4 Multivariable association framework for cross-sectional EPD-versus-LPD assessment

Applying the same collinearity control strategy (VIF < 5), PreMA was selected for final inclusion (Supplementary Table 1). The explanatory frameworks were developed incrementally: the baseline clinical model (S1) included age, gender, BMI, disease duration, and LEDD; the integrated S2 framework additionally incorporated serum DAPK1, PreMA, and PostGR (Supplementary Table 3).

**FIGURE 2:**
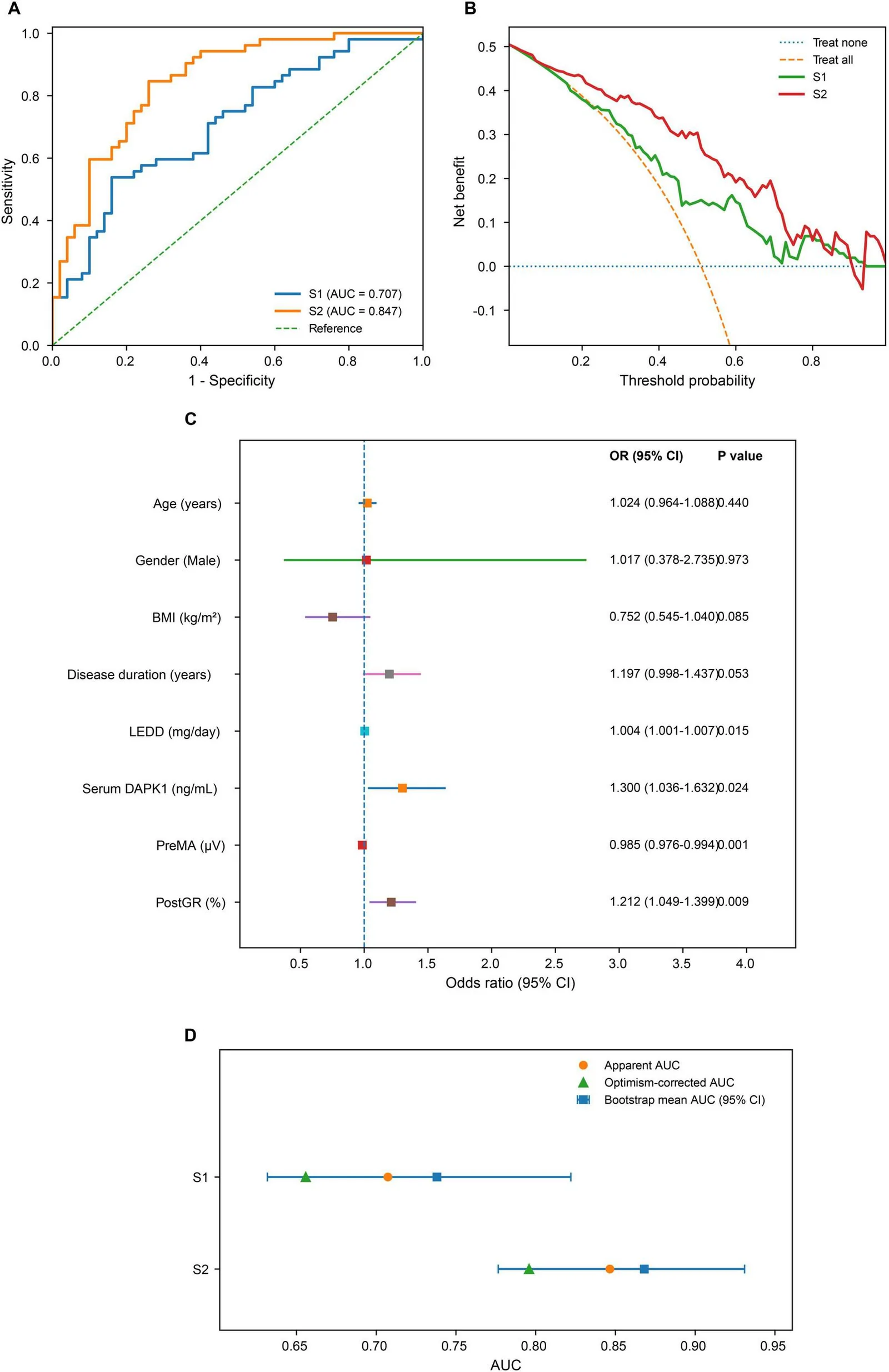
Performance and internal validation of the stepwise association frameworks for cross-sectional EPD-versus-LPD assessment. **(A)** Receiver operating characteristic (ROC) curves comparing the clinical baseline framework (S1) and the integrated multimodal framework (S2) for cross-sectional differentiation between early-stage Parkinson’s disease (EPD) and mid-to-late-stage Parkinson’s disease (LPD). S1 included age, gender, BMI, disease duration, and Levodopa Equivalent Daily Dose (LEDD); S2 further incorporated serum DAPK1, preprandial mean amplitude (PreMA), and postprandial gastric rhythm (PostGR). The apparent AUCs were 0.707 and 0.847 for S1 and S2, respectively. **(B)** Decision-curve analysis (DCA) of S1 and S2 across threshold probabilities. The S2 framework suggested a higher potential net benefit than S1 across selected threshold ranges. **(C)** Forest plot of the final integrated S2 framework, showing odds ratios (ORs), 95% confidence intervals (CIs), and *P*-values for the retained covariates. **(D)** Internal validation and optimism assessment displaying the apparent AUC, optimism-corrected AUC, and bootstrap mean AUC with 95% confidence intervals for S1 and S2 frameworks. EPD, early-stage Parkinson’s disease; LPD, mid-to-late-stage Parkinson’s disease; BMI, body mass index; LEDD, Levodopa Equivalent Daily Dose; DAPK1, death-associated protein kinase 1; PreMA, preprandial mean amplitude; PostGR, postprandial gastric rhythm; ROC, receiver operating characteristic; DCA, decision-curve analysis; AUC, area under the curve; OR, odds ratio; CI, confidence interval.

The integrated S2 framework showed improved explanatory performance compared with the clinical baseline, with a Nagelkerke pseudo-R^2^ of 0.458 and an apparent AUC of 0.847 (Figure 2A). DCA suggested potential net benefit for the S2 framework across selected threshold ranges within this cohort (Figure 2B). After adjustment for baseline clinical features and LEDD, reduced PreMA, increased PostGR, and elevated serum DAPK1 were retained as independent correlates of LPD status (all *P* < 0.05, Figure 2C and Table 4). LRT and IDI analyses indicated that adding these multimodal markers provided incremental explanatory value over the clinical baseline (LRT *P* < 0.001; IDI = 0.219, *P* < 0.001; Supplementary Table 3). Internal validation using 1,000 refit bootstrap resamples yielded a bootstrap mean AUC of 0.873 (95% CI: 0.795–0.938) and an optimism-corrected AUC of 0.795 (Figure 2D, Supplementary Figure 1B and Supplementary Table 3).

**TABLE 4:** Final integrated multivariable association model for cross-sectional EPD-versus-LPD assessment.

Predictor	OR (95% CI)	*P*-value
Age (years)	1.024 (0.964–1.088)	0.440
Gender (Male)	1.017 (0.378–2.735)	0.973
BMI (kg/m^2^)	0.752 (0.545–1.040)	0.085
Disease duration (years)	1.197 (0.998–1.437)	0.053
LEDD (mg/day)	1.004 (1.001–1.007)	0.015
Serum DAPK1 (ng/mL)	1.300 (1.036–1.632)	0.024
PreMA (μV)	0.985 (0.976–0.994)	0.001
PostGR (%)	1.212 (1.049–1.399)	0.009

Values are odds ratios (ORs), 95% confidence intervals (CIs), and *P*-values from the final integrated logistic regression model (S2). Gender was recoded before modeling as Female = 0 and Male = 1; Female served as the reference group. ORs for continuous variables represent the change in odds per one-unit increase. EPD, early-stage Parkinson’s disease; LPD, mid-to-late-stage Parkinson’s disease; BMI, body mass index; LEDD, Levodopa Equivalent Daily Dose; DAPK1, death-associated protein kinase 1; PreMA, preprandial mean amplitude; PostGR, postprandial gastric rhythm.

## 4 Discussion

This study explored cross-sectional associations between serum DAPK1 and peripheral gastric myoelectrical activity in Parkinson’s disease (Araki et al., 2021; Warnecke et al., 2022). The integrated framework was intended to evaluate whether a circulating molecular correlate and an established gastric functional readout provide complementary information across PD stage groups. These findings should be interpreted as cross-sectional associations and internally evaluated model performance, not as evidence of gastrointestinal disease origin, directional propagation, causality, longitudinal staging, or clinical implementation (Breen et al., 2019; Horsager et al., 2020).

Our multivariable analysis suggests that EPD status was associated with concurrent differences in affective burden, serum DAPK1, and postprandial gastric reactivity (PostRA). Reduced PostRA may reflect altered gastric autonomic responsiveness in early-stage disease, whereas lower PreMA and higher PostGR in LPD may reflect more prominent stage-related differences in peripheral gastric myoelectrical function (Araki et al., 2021; Oertel et al., 2024; Warnecke et al., 2022). However, these findings do not establish whether gastric dysfunction precedes, follows, or contributes mechanistically to PD progression.

Objective EGG measures may complement symptom-based assessments by providing non-invasive peripheral functional readouts (Araki et al., 2021; Wang et al., 2003). However, the observed associations should be interpreted cautiously because EGG was measured at a single standardized visit and is sensitive to motion and respiratory artifacts. The modest correlations between EGG measures and clinical scales indicate partial concordance rather than a one-to-one mapping between gastric electrophysiological abnormalities and clinical severity.

Elevated serum DAPK1 in PD may be consistent with broader inflammatory or neurodegeneration-related processes (Mansour et al., 2023; Soraci et al., 2024; Zhang et al., 2024). Experimental studies suggest that DAPK1 can phosphorylate alpha-synuclein under model conditions (Kim N. et al., 2019; Kim S. et al., 2019; Shin and Chung, 2020; Mifflin et al., 2020; Ding et al., 2025; Li et al., 2026), but serum DAPK1 in this clinical cohort cannot be taken as direct evidence of central or gastrointestinal alpha-synuclein pathology (Kelly et al., 2014). The absence of tissue-level phosphorylated alpha-synuclein validation means that the relationship between circulating DAPK1 and synucleinopathy-related pathology remains hypothesis-generating.

In the EPD-versus-LPD framework, serum DAPK1, PreMA, and PostGR remained associated with more advanced disease status after adjustment for clinical covariates and LEDD. These variables may capture aspects of systemic inflammation and peripheral autonomic dysfunction that are not fully reflected by disease duration alone. Nevertheless, the results should not be interpreted as showing that these biomarkers outperform clinical staging or that they can replace established clinical assessment. Instead, they may provide complementary cross-sectional information for characterizing clinical heterogeneity.

The combined use of DAPK1 and EGG measures improved the explanatory performance of the multivariable frameworks. The refit bootstrap analyses suggested internal stability of the integrated models, with optimism-corrected AUCs of 0.823 for E3 and 0.795 for S2. However, these findings remain internally validated and cohort-specific. Independent external validation and prospective longitudinal follow-up are required before these frameworks can be considered for clinical decision-making or progression monitoring.

Beyond individual correlations, the multivariable results support the value of combining systemic and peripheral measures to describe PD-related heterogeneity. LRT, IDI, ROC, and DCA were used to evaluate sequential model improvement and potential decision-curve benefit within the present cohort. DCA suggested potential net benefit for the integrated models across selected threshold ranges, but this should not be interpreted as validated clinical application. The lack of an independent external validation dataset remains an important limitation.

Several limitations warrant acknowledgment. First, the cross-sectional design restricts causal and temporal inference among DAPK1 elevation, EGG dysrhythmia, and clinical progression. Residual confounding cannot be excluded despite covariate adjustment. Second, DAPK1 and EGG were measured at a single time point, and serum DAPK1 was quantified using singlet ELISA because of limited clinical sample volume; these factors may introduce technical noise and cannot capture intra-individual longitudinal variability. Third, although patients underwent a 12-h medication washout for EGG evaluation, residual effects of long-acting dopamine agonists or sustained-release levodopa cannot be fully excluded; therefore, LEDD was retained as a forced covariate in the severity framework. Fourth, serum DAPK1 is not specific to PD and may reflect broader age-related or neuroinflammatory processes (Durmaz Celik et al., 2024; Tolosa et al., 2026). Future studies should include disease controls such as multiple system atrophy and progressive supranuclear palsy (Munhoz et al., 2024; Mulroy et al., 2024) and should examine correlations between serum DAPK1 and peripheral tissue phosphorylated alpha-synuclein burden. Finally, the absence of longitudinal follow-up, repeated DAPK1 assays, disease-control cohorts, and tissue-level alpha-synuclein validation limits mechanistic interpretation. Longer EGG recordings and repeated visits will also be needed to assess measurement stability.

## 5 Conclusion

In summary, this study shows that serum DAPK1 and peripheral gastric myoelectrical measures are associated with cross-sectional PD status and EPD/LPD grouping. EPD was characterized by higher affective burden, higher serum DAPK1, and reduced postprandial gastric reactivity, whereas LPD was associated with lower basal gastric myoelectrical amplitude, increased postprandial gastric rhythm, and higher serum DAPK1. These findings support an exploratory multimodal framework that may complement conventional clinical assessment by integrating systemic molecular and peripheral functional correlates. They should not be interpreted as evidence of gastrointestinal disease origin, directional gut-to-brain propagation, or causal mechanisms. Because this study was cross-sectional and lacked external validation, prospective multicenter studies with independent cohorts are required before clinical application or longitudinal staging value can be claimed.

## Data Availability

The raw data supporting the conclusions of this article will be made available by the authors, without undue reservation.
