# Supplementary material for: Multimodal integration of serum DAPK1 and gastric myoelectrical activity reveals stage-specific correlates in Parkinson’s disease
Source: Front Aging Neurosci. 2026 Aug 7;18:1866257. doi: 10.3389/fnagi.2026.1866257 (PMC13493592; doi:10.3389/fnagi.2026.1866257)
Supplement: Supplementary file 1 [file Data_Sheet_1.docx]

**Multimodal Integration of Serum DAPK1 and Gastric Myoelectrical Activity Reveals Stage-Specific Correlates in Parkinson’s Disease**

Zhining Li ^1,2^,*, Pei Lu ^3^,*, Can Li ^2^, Liangqun Rong ^2^, Zixuan Wu ^3^, Yumeng Liu ^3^, Yuyao Tian ^4^, Aiping Gong ^2^, Lishun Xiao ^3,#^, Chunfeng Liu ^1,#^

1 Department of Neurology and Clinical Research Center of Neurological Disease, The Second Affiliated Hospital of Soochow University, Suzhou, Jiangsu, 215004, China

2 Department of Neurology, The Second Affiliated Hospital of Xuzhou Medical University, Xuzhou, Jiangsu, 221006, China

3 School of Public Health, Xuzhou Medical University, Xuzhou, Jiangsu, 221004, China

4 Department of Biomedical Engineering, The Chinese University of Hong Kong, Shatin, N.T., Hong Kong SAR

* These authors have contributed equally to this work and share first authorship.

\# Corresponding authors: No. 209 Tongshan Road, Xuzhou Medical University, Xuzhou, Jiangsu Province, 221004, China (Lishun Xiao, E-mail: xiaolishun@xzhmu.edu.cn); No. 1055 Sanxiang Road, Gusu District, Suzhou, Jiangsu Province, 215004, China (Chunfeng Liu; E-mail: liuchunfeng@suda.edu.cn; TEL: 00 86 512 67783307).

**Supplementary Table S1. Multicollinearity diagnostics for candidate variables and final model blocks**

| **Block** | **Variable** | **Tolerance** | **VIF** | **Diagnostic decision** |
| --- | --- | --- | --- | --- |
| Initial candidate block | Age (years) | 0.861 | 1.162 | Retained |
| Initial candidate block | Gender (Male) | 0.928 | 1.077 | Retained |
| Initial candidate block | BMI (kg/m²) | 0.921 | 1.086 | Retained |
| Initial candidate block | Disease duration (years) | 0.822 | 1.216 | Retained |
| Initial candidate block | LEDD (mg/day) | 0.888 | 1.126 | Retained |
| Initial candidate block | Global-Cog (MoCA) | 0.791 | 1.265 | Retained |
| Initial candidate block | Mood Score | 0.806 | 1.240 | Retained |
| Initial candidate block | Serum DAPK1 (ng/mL) | 0.843 | 1.186 | Retained |
| Initial candidate block | PostGR (%) | 0.676 | 1.479 | Retained |
| Initial candidate block | PostRA (μV·s) | 0.020 | 50.709 | Retained preferentially after excluding collinear counterpart |
| Initial candidate block | PostMA (μV) | 0.022 | 45.890 | Excluded from final models: highly collinear with PostRA |
| Initial candidate block | PreMA (μV) | 0.044 | 22.856 | Retained preferentially after excluding collinear counterpart |
| Initial candidate block | PreRA (μV·s) | 0.040 | 24.830 | Excluded from final models: highly collinear with PreMA |
| Final E3 block | Age (years) | 0.924 | 1.083 | Final retained; VIF < 5 |
| Final E3 block | Gender (Male) | 0.938 | 1.066 | Final retained; VIF < 5 |
| Final E3 block | BMI (kg/m²) | 0.973 | 1.028 | Final retained; VIF < 5 |
| Final E3 block | Mood Score | 0.684 | 1.463 | Final retained; VIF < 5 |
| Final E3 block | Global-Cog (MoCA) | 0.724 | 1.382 | Final retained; VIF < 5 |
| Final E3 block | Serum DAPK1 (ng/mL) | 0.912 | 1.097 | Final retained; VIF < 5 |
| Final E3 block | PostRA (μV·s) | 0.904 | 1.106 | Final retained; VIF < 5 |
| Final S2 block | Age (years) | 0.921 | 1.086 | Final retained; VIF < 5 |
| Final S2 block | Gender (Male) | 0.979 | 1.022 | Final retained; VIF < 5 |
| Final S2 block | BMI (kg/m²) | 0.981 | 1.020 | Final retained; VIF < 5 |
| Final S2 block | Disease duration (years) | 0.904 | 1.106 | Final retained; VIF < 5 |
| Final S2 block | LEDD (mg/day) | 0.934 | 1.071 | Final retained; VIF < 5 |
| Final S2 block | Serum DAPK1 (ng/mL) | 0.982 | 1.018 | Final retained; VIF < 5 |
| Final S2 block | PreMA (μV) | 0.922 | 1.085 | Final retained; VIF < 5 |
| Final S2 block | PostGR (%) | 0.984 | 1.016 | Final retained; VIF < 5 |

**Note:** Variance inflation factor (VIF) diagnostics were used to evaluate multicollinearity. A conventional exclusion threshold of VIF > 5 was applied. The initial candidate block identified collinearity between PostRA and PostMA and between PreMA and PreRA. PostRA and PreMA were preferentially retained, while their collinear counterparts were excluded. Final E3 and S2 model blocks showed VIF values below 5 for all retained variables.

**Supplementary Table S2. Stepwise multivariable association frameworks for cross-sectional HC-versus-EPD assessment**

| **Variable** | **E1** | **E2** | **E3** |
| --- | --- | --- | --- |
| Age (years) | 1.011 (0.971-1.053); 0.587 | 0.995 (0.948-1.045); 0.837 | 1.023 (0.969-1.079); 0.410 |
| Gender (Male) | 1.111 (0.558-2.212); 0.765 | 0.803 (0.354-1.821); 0.599 | 0.945 (0.397-2.250); 0.898 |
| BMI (kg/m²) | 1.200 (0.971-1.484); 0.092 | 1.292 (1.012-1.649); 0.040 | 1.267 (0.979-1.641); 0.073 |
| Mood Score | - | 1.191 (1.085-1.307); <0.001 | 1.170 (1.058-1.294); 0.002 |
| Global-Cog (MoCA) | - | 0.869 (0.758-0.997); 0.045 | 0.881 (0.767-1.011); 0.071 |
| Serum DAPK1 (ng/mL) | - | - | 1.368 (1.053-1.777); 0.019 |
| PostRA (μV·s) | - | - | 0.982 (0.970-0.994); 0.004 |
| Nagelkerke Pseudo-R² | 2.85% | 36.62% | 46.00% |
| AUC (apparent) | 0.608 | 0.798 | 0.851 |
| AUC (bootstrap mean, 95% CI) | 0.628 (0.534-0.730) | 0.812 (0.718-0.892) | 0.862 (0.791-0.925) |
| Optimism-corrected AUC | 0.556 | 0.772 | 0.823 |
| LRT vs previous | - | 43.019 (df=2); <0.001 | 14.505 (df=2); <0.001 |
| IDI vs previous | - | 0.280; P <0.001 | 0.072; P = 0.002 |

**Note:** Values are OR (95% CI); P unless otherwise indicated. E1 included age, gender, and BMI. E2 added Mood Score and Global-Cog (MoCA). E3 added serum DAPK1 and PostRA. Bootstrap mean AUCs and optimism-corrected AUCs were estimated by refitting the model across 1,000 bootstrap resamples and are provided for internal validation only. LRT, likelihood ratio test; IDI, integrated discrimination improvement; DAPK1, death-associated protein kinase 1; PostRA, postprandial reaction area.

**Supplementary Table S3. Stepwise multivariable association frameworks for cross-sectional EPD-versus-LPD assessment**

| **Variable** | **S1** | **S2** |
| --- | --- | --- |
| Age (years) | 1.027 (0.974-1.081); 0.324 | 1.024 (0.964-1.088); 0.440 |
| Gender (Male) | 0.884 (0.378-2.066); 0.776 | 1.017 (0.378-2.735); 0.973 |
| BMI (kg/m²) | 0.833 (0.641-1.082); 0.171 | 0.752 (0.545-1.040); 0.085 |
| Disease duration (years) | 1.256 (1.068-1.477); 0.006 | 1.197 (0.998-1.437); 0.053 |
| LEDD (mg/day) | 1.002 (0.999-1.004); 0.157 | 1.004 (1.001-1.007); 0.015 |
| Serum DAPK1 (ng/mL) | - | 1.300 (1.036-1.632); 0.024 |
| PreMA (μV) | - | 0.985 (0.976-0.994); 0.001 |
| PostGR (%) | - | 1.212 (1.049-1.399); 0.009 |
| Nagelkerke Pseudo-R² | 18.59% | 45.76% |
| AUC (apparent) | 0.707 | 0.847 |
| AUC (bootstrap mean, 95% CI) | 0.742 (0.644-0.829) | 0.872 (0.795-0.938) |
| Optimism-corrected AUC | 0.654 | 0.795 |
| LRT vs previous | - | 27.556 (df=3); <0.001 |
| IDI vs previous | - | 0.219; P <0.001 |

**Note:** Values are OR (95% CI); P unless otherwise indicated. S1 included age, gender, BMI, disease duration, and LEDD. S2 added serum DAPK1, PreMA, and PostGR. Bootstrap mean AUCs and optimism-corrected AUCs were estimated by refitting the model across 1,000 bootstrap resamples and are provided for internal validation only. LRT, likelihood ratio test; IDI, integrated discrimination improvement; DAPK1, death-associated protein kinase 1; PreMA, preprandial mean amplitude; PostGR, postprandial gastric rhythm.

**Supplementary Figure S1. Bootstrap AUC distributions for the final integrated frameworks.**

**Note:** (A) Distribution of bootstrap AUCs for the final integrated E3 framework in the HC-versus-EPD analysis. The dashed vertical line indicates the apparent AUC (0.851), and the solid vertical line indicates the bootstrap mean AUC (0.862) estimated from 1,000 refit bootstrap resamples; the bootstrap 95% CI was 0.791–0.925. (B) Distribution of bootstrap AUCs for the final integrated S2 framework in the EPD-versus-LPD analysis. The dashed vertical line indicates the apparent AUC (0.847), and the solid vertical line indicates the bootstrap mean AUC (0.872) estimated from 1,000 refit bootstrap resamples; the bootstrap 95% CI was 0.795–0.938. These results represent internal validation and should not be interpreted as external validation.
